# The Nursing Supplement

**Published:** 1888-07-07

**Authors:** 


### The Nursing Mirror.

All Contributions for this Supplement should be addressed "The Nursing Mirror," care of The Editor, 38, Old Jewry, E.C.

### En Passant.

A BRAVE NURSE.—We are delighted to hear that Nurse Finnis, whose heroic conduct met with such approval lately, has not suffered from her unselfish deed, but is enjoying good health. Those captious critics who find fault everywhere, and accused Nurse Finnis of encountering unwarrantable risk, may hide their diminished heads. On the authority of the matron of the hospital we learn that Nurse Finnis did not suck the tube, but blew violently down it and so cleared it; nor was her devotion only momentary. For two days and nearly two nights she scarcely left the child's side, and did all she could to prolong life. Hers was the heroism which faces long days of little duties, and yet does not fall short when a great deed is demanded. There are few of whom the same can be said, and those who are disposed to cavil as to the right of facing such risk, had better consider and be silent.

NEWS FROM SHEFFIELD.—Miss Mary E. Corvan has taken possession of "Westholme," a pleasant house and garden where most of her nurses soon hope to join her. Thirty-one have sent in their notices to the committee, in spite of a carefully-worded letter—partly threatening, partly promising, which was sent to each nurse by the chairman. For some time after her rude expulsion, Miss Corvan was quite ill with worry, but now her sister from Taunton has joined her, and they are working happily together. We learn from a private source that Miss Corvan never asked her nurses to go with her, nor canvassed for cases till after she left the institute; so what the conspiracy was of which the committee accused her it is impossible to divine.

A NURSE'S BREACH OF PROMISE.—In the Queen's Bench Division last week, before Mr. Justice Charles, the case of Welsh v. Reeks was heard. This was an action to recover damages for the breach of a promise to marry; but the usual course in such cases was reversed, the plaintiff being a man and the defendant a woman. Last year the plaintiff was a grocer's assistant at Surbiton, and in April he had to go into the Cottage Hospital there. The defendant was nurse in that hospital, and she generally had charge of the plaintiff. Very soon there were some passages of affection between them; but it was not until Jubilee Day, June 21st, that there was any definite promise of marriage. The plaintiff was 24 years of age, the defendant was some four or five years older; and it was said the defendant was more the wooer than the wooed, and that in one of her letters she said she "had been seeking his friendship more than he hers." Anyway, on further aquaintance the nurse no longer loved the grocer's assistant, and told him so, and demanded back her letters. He wrote, "I ask you to let me keep your letters in remembrance of happier days," and afterwards wrote saying that she could have the letters "by paying for them." Again he wrote that he expected "£50 for the heartless manner in which I was thrown over by Miss Reeks." For the defence it was said that the defendant was in such a condition of health that she could not appear in court. It was further said that her income at Surbiton was 10*s.* a week with board and lodging. This was after she left the Cottage hospital and had become a parish nurse. His Lordship in the end gave judgment for the defendant, upon the ground that she had been exonerated by the plaintiff from the performance of her promise.

ACTRESS AND NURSE.—We all know how generous actors and actresses are in giving matinées in the cause of charity, but the artistic side of their nature generally keeps them far from sights of sorrow. It is told, however, of Fraulein Bertha Feldan, a well-known German actress, that when cholera broke out in the small Hungarian town where she was born, she went immediately to the plague-stricken streets, and nursed the sick and dying night and day. There was some heroism in this, for the panic caused by the cholera was so great that few would venture into the dirty little houses to attend to the poor stricken inmates. The end of the story is more amusing than pathetic: "Bertha," said an old priest as he blessed her, "you have a tender heart; much will be forgiven you because of your goodness." He evidently thought that an actress must necessarily have many sins to expiate.

DIET OF NURSES.—In a letter to *Nursing Notes* Miss Twining makes a few sensible and forcible remarks on the diet of nurses. It will be remembered by many of our readers that this subject was fully discussed before The Hospitals Association about three years ago, and that the conclusion arrived at was that improvement in the diets was greatly to be desired. Miss Twining maintains that this improvement appears not, but perhaps Miss Twining is thinking more of workhouse infirmaries than hospitals. In many of our large hospitals the food now provided for nurses leaves very little to be desired; in nearly every country infirmary it leaves a great deal to be desired! The nurses are often there expected to cook and eat very indifferent food, and wash up the utensils in half-an-hour. In the hospitals, Miss Twining says the chief fault is the lack of fresh vegetables and fruit, and the unpalatableness of the cold fish or pudding so often provided for supper. This is certainly a subject which should be kept before the public till doctors and matrons are as careful over the diet of the nurses as they are over that of the patients.

THE NIGHTINGALE FUND.—Last year's report of the working of this fund has been issued, and shows a steady advance as usual. During the year 49 probationers were admitted, of whom 15 resigned or were discharged, 31 completed their training, and there were 32 left in the school at the beginning of the year. The annual gratuity of £2 allowed by the regulations, for the term of three years, to the certified nurses who have completed a year's work satisfacfactorily either in hospital or district nursing, was awarded to 76 nurses. The appointments gained by St. Thomas's nurses during the past year include the matronships of Folkestone Hospital, Hospital for Incurables, Donnybrook, South Hants Infirmary, Cheltenham Hospital, and Ealing Cottage Hospital. Sisters and head nurses were also supplied to many institutions. This is the first report drawn up by Miss Pringle, the worthy successor of Mrs. Wardroper; and it is pleasant to see that Miss Pringle highly approves of the arrangements of the Home, and appreciates Miss Crossland's powers of instructing probationers. It is well known how thorough is the teaching received by Nightingale nurses, and this is doubtless the reason why they receive such good appointments. The clinical lectures delivered by Dr. Bristowe and Dr. Ord to the senior and special probationers are of remarkable value as being a form of instruction to nurses too often neglected. During the year there were over a thousand applications for the rules of admission to the training school, but many of the applicants were either too young or too old. Ordinary probationers should enter between the ages of twenty-five and thirty-five, and lady probationers between the ages of twenty-six and thirty-six. The regulations as to the admission of candidates of both classes can be obtained by writing to Miss Pringle.

### The National Pension Fund for Nurses.

All Communications should be addressed The Editor, The Lodge, Porchester Square, W.

An esteemed provincial physician writes to point out that it is generally understood that the attacks upon this fund, which have appeared in one or two quarters, are inspired by personal pique. He refers to the article which appeared in the "Journal" on May 6th last, and asks us to restore confidence by proving that the rates are not excessively high, and to publish the opinion of an independent actuary upon them. We have already done this in the "Journal" (pp. 811, 815, and 983), where it is pointed out that this fund is managed gratuitously by some of the most able and representative City authorities, including Lord Rothschild, Mr. Henry Hucks Gibbs (late governor of the Bank of England), Mr. E. A. Hambro and Mr. Clifford Wigram (directors of the Bank of England), Mr. Walter H. Burns (of Messrs J. S. Morgan and Co.), Mr. Alfred de Rothschild, and Mr. Edward Rawlings, as well as by eminent medical men and hospital managers, including Sir Edmund Currie, Major Ross, M.P., Dr. Bristowe, F.R.S., Mr. Thomas Bryant, Mr. R. Brudenell Carter, and Dr. Steele, of Guy's Hospital; that the fund commences with a bonus income of about £1,000 per annum; that generous donors have provided the deposit of £20,000; that the fund is a mutual fund, and that, assuming the rates are higher than necessary, which they are not, the full value of each nurse's money entrusted to the managers will be returned to her when her pension fall in, because the pensions stated are the lowest amounts payable irrespective of bonus additions, whilst those quoted by insurance offices are the highest sums which will be paid under any circumstances. Indeed, the rates are from 7 to 10 per cent. lower, as we have already pointed out, than the Post Office rates, which will shortly be raised in consequence of the conversion of consols from 3 to 2¾ per cent.

The unsoundness and absurdity of the comparisons made between the Pension Fund rates and those nominally offered by a first-class office like the Prudential is well proved by Mr. George King, the actuary of the fund, who has ascertained from the actuary of the Prudential that his company has been doing business in immediate annuities at a loss, and that the whole of the annuity tables of that office are about to be revised. The truth is that those who have criticised the rates offered to nures by the managers of this fund must be either ignorant or ill-disposed. As a matter of fact no man of business dare advise a nurse to invest her small savings in any office which undertook to do deferred annuity business at lower rates than those offered by the Pension Fund.

The statements which have been published and are being circulated, intimating that the payments required are, from an actuarial point of view, excessive, have also been effectually refuted by Mr. King, and by independent evidence, and the comparisons therein made are based upon palpable error. This is admirably proved in the address of Dr. Bristowe to the members of the Hospitals Association, wherein he gives the opinions and verdict of an eminent and independent actuary, to whom he had referred the Pension Fund rates. Copies of Dr. Bristowe's address may be obtained on application to the Secretary of the Fund, 38, Old Jewry, E.C.

It is gratifying to read in the published statement that the success of this fund is now assured; 120 policies have already been accepted, 326 proposals have been received, and 1,600 nurses have applied at the office for forms of proposal to fill up, whilst similar applications are being made in increasing numbers every week. We congratulate the promoters upon this success, knowing as we do that the scheme is absolutely sound, that the *bona fides* of its promoters is unassailable and undoubted, and that the financial strength of the National Pension Fund can be classed with that offered by the British Funds and the Bank of England.

No such opportunity can again be anticipated by women of the nursing profession of providing for themselves by reasonable and moderate payments. The medical profession will, therefore, we are convinced, not fail to impress upon those nurses with whom they come in contact the duty of self-help, and the importance of availing themselves of the thoughtful munificence and well-devised administration which are now provided by this fund on their behalf. The feeling of indignation against the reckless and unthinking opponents of this noble undertaking, to which our correspondent gives expression, is manifesting itself in many places throughout the country; and medical officers—honorary and resident—as well as laymen and officials, are taking opportunities of bringing the matter under the notice of the nursing staff of the institution to which they are attached. Further, the responsible managers of the great London hospitals are arranging a conference to determine how they can best help their nurses to join the fund. They could render no greater service. In some instances inquiry will show that the net emoluments of the nurses do not amount to £15 per annum, as is the case at the London Hospital, the largest in the country, owing to the extra expenses devolving upon the nurses there, each of whom has to provide herself with washing and with other things which it should be the first duty of the managing body to provide free of cost.

We cannot believe that the London Hospital Committee, the Chairman of which is also Chairman of the General Purposes Committee of this fund, will consent to permit the present unsatisfactory and unjust arrangement to continue. We trust that it will promptly consent to pay its nurses at any rate as much as is paid to those who labour in poor-law infirmaries. In any case, it is the duty, as we feel sure it will be the pleasure, of medical men and hospital managers to interest themselves in the Pension Fund, and to use their utmost endeavours to secure that every nurse with whom they come into communication shall at once, or as speedily as possible, avail herself of the great advantages and protection which this fund affords to the whole nursing body.—*British Medical Journal.*

### Notes from America.

Nurses and Politics.—We are all aware that journalism in America appreciates sensational headings; still, the following will surely strike our readers as amusingly strange: "The Training School for Nurses. Four Ministering Angels graduated. Addresses by the Faculty." Then follows an account of the training school which is in connexion with the Women's Hospital, Chicago, and has a diet kitchen, where each nurse is trained. This looks as though cookery were regarded as a chief point in nursing education, and we advise the young man who advertises in our pages as "able to make messes for the sick" to turn his attention westwards. The ministering angels are described as young women wearing a neat uniform—blue and white seersucker dress, spotless apron, and mob cap. Another American paper has an account of a training school at Illinois. This school seems to have done good service, for before its foundation in 1880 the nurses of the Hospital were all either women of bad character or convalescent patients; and the respectable people preferred to suffer and die in their own homes rather than be subjected to abuse and neglect at the hospital. In 1885 the physicians called attention to a very marked decrease in the death-rate in the hospital, owing to the better nursing. But perhaps the phrase in this report which will most astonish our English ears is the following: "Most of the gentlemen of the board were politicians, and not disposed to view favourably any plan tending to decrease their patronage." Fancy nurses being appointed by political patronage!

### Everybody's Opinion.

*[Correspondence on all subjects is welcome, but correspondents would oblige by writing on one side of the paper only.]*

### NURSES AND PATIENTS.

LETTERS on the subject of James Macfarlane's opinions continue to come in. Here we give the remarks of a Patient, a Nurse, and a Student:—

ANOTHER PATIENT SPEAKS.

SUPPOSING that a large number of your readers are "heads of families," and others residing at a distance from the larger hospitals, and dependent to a certain extent on patients for information respecting life in hospital, I should like to subject a few remarks for the perusal of those who may have read a contribution from your correspondent, "James Macfarlane," on the 11th inst. As a patient who has spent six months in one of the largest London hospitals, besides possessing a fair knowledge of other such institutions, and being acquainted with a number of patients, my evidence may be considered equal to that of an ordinary individual. Whether this be so or not, I utterly disagree with your correspondent in his opinion of nurses, and have no hesitation in saying that his opinions are not shared by the majority of patients. During my prolonged stay in hospital I had ample opportunities of watching carefully the lives of nurses and patients in both medical and surgical wards; and as for nurses being "hard, and careless of our feelings," my experience was certainly the very opposite—we were all treated most kindiy and carefully. People as a rule do not seem able to understand the difference between a nurse that is hard and one who may be most kind, yet able to control her feelings under all circumstances. I have repeatedly noticed that the latter accomplishment (which nurses only acquire by careful training) is too often mistaken for "hardness." And is not this self-control one of the most desirable characters in a nurse? Should not we patients soon cry out if a nurse showed signs of disgust or irritation when attending offensive or irritable cases? I think so. And if we have self-control in one case we must expect it in another. It is well known that to some patients sympathy as usually understood would be absolutely injurious; and people's opinions differ too as regards kind ness. We are many men with many minds; the variety of human nature one meets with in hospital life is quite sufficient evidence to convince one that for a nurse to be on good terms with all her patients is a task of no small dimensions. Now, if as stated, nurses become hardened, careless, and unsympathetic from seeing so much suffering, we have every reason to suppose that doctors, students, and sisters become likewise; and if our hospitals are attended by such inhuman monsters, one can hardly help expressing surprise that the death rates of the various institutions are not higher, or that a patient can ever be induced to subject himself to the treatment of such places. Fortunately though, this state of things does not exist; those who tend us in our hospitals know the pains we suffer, and the discomforts we have to go through, and are consequently better able to sympathise with us than those who do not possess such knowledge. But as it is a nurse's mission to nurse and not to sympathise, after the manner of certain well-meaning people, we must not expect her to show her sympathy in any conspicuous way. If people were better acquainted with the life or a nurse in a large hospital, many would be at a loss to know how it is that there is always a supply of those who are willing to devote their lives for the alleviation of our sufferings. Working some twelve or fourteen hours per day under severe discipline, always subject to visits from their many superiors—often at unexpected times — who have extremely sharp eyes, is it any wonder that a nurse is a little irritable or abrupt at times? If all the machinery works smoothly, she can usually wade through her many duties successfully. But unforeseen things will happen even in hospitals, and when they do it is pretty certain that doctor, matron, or sister, sometimes all three, will appear on the scene. Perhaps an unexpected case will come in requiring immediate attention, or a patient may have a fit of hæmorrhage, as if for the occasion. The result of even one of these occurrences, when "things will not go right," is, to say the least of it, trying. I regret being incapable to reply to the matrimonial part of your correspondent's letter, and can only suggest that he seems to have mistaken your columns for those of certain journals devoted to that subject.

Bournemouth. FREDERIC J. TANNER.

A WORD FROM NORTH WALES.

ANENT the letter appearing in your last issue, signed "James Macfarlane," I should like to say the class of patients which he represents is well known and much avoided by all nurses of hospital experience as being weak-minded and sentimental, expecting us to combine the duties of a trained nurse with those of a wife, without any present or future responsibility resting on him. This makes his concluding remark on the choice of a wife very galling. We should not like any one to think so badly of us as to suppose we should feel anything but humiliation at an offer from a man of his stamp. A NURSE OF TEN YEARS' EXPERIENCE.

THE STUDENT SPEAKS.

JAMES MACFARLANE has caught it hot, still I feel bound to say a word on his side. The tone of self-satisfaction and approbation which the "Mirror" takes up with regard to nurses is likely to foster all sorts of faults in them. It is just as well to acknowledge that all nurses are not angels, and that every nurse might improve if she tried. I have known many nurses far from perfect, and they hold such responsible posts that they ought to be as near perfection as possible. The life of a nurse is not so hard that all should be lauded who enter it. Many of them have their own ends in view, as is only natural. MEDICAL STUDENT.

### THE PAY OF PRIVATE NURSES.

IN reference to "Islands'" paragraph, I was not aware that any institution paid so little as £20 per annum to a qualified nurse for private work; though, of course, many nurses have to attend cases during their term of training when they may be receiving even less than that amount of salary. My experience of private nursing is that the work is by no means heavy, as a rule; in fact, after giving it a good trial, I have returned to hospital work as being more active, and consequently more to my taste. I believe in some institutions, when a nurse has had a heavy or risky spell at a private case, she does get some extra pay, although it may not be a standing rule. I think my experience must have been more fortunate than you correspondent's; for on two occasions (after nursing heavy cases of diphtheria and small-pox respectively) the matron of the home to which I was attached paid me over and above the rate of salary she had agreed to do, and we could always rely on her sympathy with us in any difficulties we might have to contend with. On returning from an infectious case we went into quarantine in a cottage kept for that purpose for whatever the length of time the period of latency of the disease we had been attending might be. A private nurse does get her rest disturbed, but I have found medical men most considerate on that point, in giving the family to understand that a nurse requires rest and outdoor exercise, and must have it if her work is to be done efficiently. You certainly meet with ignorant people sometimes amongst your cottage cases, though my happiest time since I have been a nurse was three months when I was nursing some cottage cases of small-pox, and the gratitude you earn from such patients is far sweeter and more gratifying than any pay an institution can render you. T. A.

### A PROBATIONER'S PROTEST.

May I add a few words to "A Probationer's Protest"? I went to —— Hospital for six months' improvement, having been already over six months working in a small place; and having only leave for one year's training, I was anxious to learn "nursing," and *paid so much* to do so. Instead of nursing, I spent most of my time doing up five firesides, cleaning sinks, and washing up. Our hours were long: we began work at a quarter to eight, having risen at half-past six, the time between being spent in breakfast, prayers, and cleaning up my room—if room it could be named, where there was not room to walk, except with one foot on my bed and one on my big box. It was part of the *servants' cubicle!*—no palliasse, only a thin mattress and flock pillow. I was also put into dirty sheets, the room and bedding swarmed with fleas, of which I killed dozens, having dazed them first with camphor. We had ten minutes allowed for dinner, half-an-hour for tea. At about a-quarter to ten we went to our bed-rooms, worn out, as we were never allowed to sit down, even if our work was done; neither might we read any but medical books, or do any knitting, &c., therefore there was no inducement to get finished one moment before the time. We had two hours off every other day, and to church every other Sunday. I was very ill, with a temperature of 103 deg., and was told I ought to feel honoured by the house surgeon coming up to see me. And when a friend (for whom I had got a fellow-probationer to write) came to see me, I was dragged out of bed and taken to a sitting-room—I suppose for fear she should sit down on the only chair there was—which was without a seat. My health broke down, and I left at the end of my month. I know how to do house-work; but as I paid, I was very indignant at being so unfairly treated and roughly used. Q.

### UNPLEASANT DUTIES.

Our night nurses come on duty at half-past nine, and, if they can, do the ward washing in the night. If they are too busy to get it done (as often happens), they have to do it either before or after their half-past nine a.m. dinner. "Doing" the linen means hauling it over to find the marks, folding it with marks out, counting it, and putting away. The hauling business is very unpleasant, and we want to know if there is not some better arrangement in other hospitals.

Two Night Nurses.

### Notes and Queries.

To Correspondents.—1. Questions may be written on post-cards. 2. Advertisements in disguise are inadmissible. 3. In answering a query please quote the number. 4. If a private answer is desired, a stamped addressed envelope must be forwarded.

(19) *St. Katherine's Hospital*—Information wanted about this hospital and its work; and how the Queen's Nursing Fund scheme is to work in connexion with it?

(26) *South Africa.*—Information wanted as to how to secure a post as nurse in South Africa.—*Miss K.*

(28) *Home Wanted*, for an incurable epileptic.—*H. Austin.*

(34) *Undenominational Sisterhood.*—Is there any community of women working together at practical charity, but under no distinct religious system, each following her own conscience?—*A.H.*

### Answers.

(33) *Adita* is advised to apply to the Midwives' Institute, 15, Buckingham Street, Strand.

*W. H. T.*—Application should be made, by letter or personally, to the Secretary at the College.

(32) *Agnes Jones.*—Nurse Frances is informed that the "Memorials of Agnes Elizabeth Jones," by her sister, is published by Strahan and Co., 34, Paternoster Row.

### Vacancies.

The following vacancies are announced:—

### Matrons.

Warneford Hospital, Leamington, Spa; £60.

Shrewsbury Infirmary; £100.

Swindon Victoria Hospital.

### Nurses.

Worcester Institute; several.

Blackburn Infirmary; £27.

Brompton Consumption Hospital; two.

Suffolk Hospital, Bury St. Edmund's; £20.

Southport Nurses' Home.

Kingston Home, Hull.

Eccles and Patricroft Hospital. £20.

Glasgow Sick Poor and Private Nursing Association.

Newport Infirmary, Mon.; two.

Swansea and South Wales Nursing Institute; two.

Cancer Hospital (Free), Brompton; several.

Ipswich Nurses' Home; several.

Shoreditch Infirmary; several.

### Probationer.

Hayward Hospital, Burslem

### Appointments.

Central Ophthalmic Hospital.—We regret that we should have made a slight mistake in our notice of Miss Howard Jones's appointment to the matronship of the Central Ophthalmic Hospital. Miss Howard Jones spent only six weeks at the Grosvenor Hospital, not nine months, as we erroneously stated. It speaks all the more highly for her qualifications, however, that she should in so short a time have won such a high esteem on the part of all she came in contact with there, as her testimonials from the officials of the "Grosvenor" show.

Sheffield Nurses' Institute.—Miss Armstrong has been appointed matron of the Sheffield Nurses' Institute. Miss Armstrong was trained at the Liverpool Royal Infirmary, and worked many years in the Rochdale Hospital. Latterly she has been helping the Deaconess Institute at Chester.

The London Hospital.—Miss Pratt and Miss Alice Smith have been respectively appointed sisters of Rowsell and Blizard wards of the London Hospital. Miss Pratt was lately Nurse Emily in the same hospital, and Miss Smith finished her period of training by passing out well at the examination.

Glamorgan and Cardiff Infirmary.—Mrs. A. M. Francis has been appointed matron of the Glamorgan and Cardiff Infirmary. Though Mrs. Francis has not recently been practising her profession, her long and high record of work amply justifies the Infirmary committee in their selection of her as successor to Miss Pratt. Mrs. Francis was trained at St. George's Hospital, which she left to become sister-in-charge at the Nottingham General Hospital. Thence she went to the Queen's Hospital, Birmingham, as lady superintendent of nurses, and finally, after a stay of three and a-half years, became matron of Chester General Infirmary. She remained there rather more than four years, leaving in December, 1881, to be married. Mrs. Francis has recently become a widow, and is therefore anxious to resume the exercise of her profession. We congratulate her on having found a post where her capacities will have full scope. The committee are exceedingly fortunate in having secured Mrs. Francis as matron.

Withington Workhouse Hospitals.—Miss Sibly has been appointed matron to the Workhouse Hospitals, Withington, which contain nearly 1,000 beds. Miss Sibly was trained at the Liverpool Workhouse, where the late Miss Jones did so great a work. She remained there for four years, and underwent a thorough training in the medical, surgical, and fever wards. She not only acquitted herself honourably in the final examinations for her certificates, but obtained a prize for note-taking in Dr. Alexander's department of the hospitals. She has acted as housekeeper, night superintendent, and assistant matron at Liverpool or St. Mary Abbott's Infirmary, with great credit to herself and to the entire satisfaction of the authorities. Miss Sibly has devoted herself to nursing, and her promotion, which is justly merited, cannot fail to gratify her many friends.

### Examination Questions.

The following are questions given to probationers in the Brooklyn Hospital, New York:—

1st. Name the most important organs of circulation, of respiration, of digestion. Name all the urinary organs.
2nd. Name all the bones of the head.
3rd. Name the four classes of bones and give an example of each.
4th. Name all the carpal bones.
5th. State the exact position of the stomach.
6th. Describe the course of the blood from the right auricle to the left ventricle.
7th. What change takes place in the blood while in the lungs?
8th. Describe the process of digestion which takes place in the stomach.
9th. Where does digestion begin? Where terminate?
10th. What is the normal specific gravity of urine?
11th. For what is digitalis used chiefly, and what is the dose of the tincture?
12th. Give another name for brandy; for whiskey; for alcohol; for prussic acid; for muriatic acid.
13th. State the usual dose of quinine sulphate; of tincture ferri chloridi; of oleum morrhuae.
14th. Name three simple emetics.
15th. What is meant by the words tincture, infusion, diuretic, analgesic, mydriatic?
16th. What position would a patient be likely to assume, suffering from peritonitis? from colic? from pluerisy?
17th. Define the terms dyspnoea and aphonia.
18th. State the normal temperature, pulse and respiration.
19th. Describe a dicrotic pulse.
20th. In what diseases is it particularly necessary to disinfect the excrement?

